# AHNAK2 Urinary Protein Expression as Potential Biomarker for Bladder Cancer Detection: A Pilot Study

**DOI:** 10.5152/tud.2022.22132

**Published:** 2022-11-01

**Authors:** Selim Komina, Gordana Petrusevska, Rubens Jovanovic, Slavica Kostadinova Kunovska, Sotir Stavridis, Saso Dohcev, Skender Saidi, Sonja Topuzovska

**Affiliations:** 1 Ss. Cyril and Methodious University, Faculty of Medicine, Institute of Pathology, Skopje, North Macedonia; 2 Ss. Cyril and Methodious University, Faculty of Medicine, University Urology Clinic, Skopje, North Macedonia; 3 Ss. Cyril and Methodious University, Faculty of Medicine, Institute of Medical and Experimental Biochemistry, Skopje, North Macedonia

**Keywords:** AHNAK2, biomarker, bladder cancer, ELISA

## Abstract

**Objective:**

This study aimed to measure the AHNAK2 urinary levels in bladder cancer patients.

**Material and methods:**

This prospective case–control study enrolled 67 participants between January and March 2019 and were categorized into bladder cancer group (n = 37), with histologically proven bladder cancer, and control group (n = 30), with histologically verified benign lesions or with no bladder cancer indication during follow-up. Urine samples of 15 mL were collected in the mid-morning before cystoscopy/surgery and an enzyme-linked immunosorbent assay was performed as per the manufacturer’s protocol. Bladder malignancies were classified according to the World Health Organization Tumor Classification. Group’s associations were evaluated with the Student *t*-test, Spearman’s rank correlation, and Mann–Whitney *U* test, while receiver operating curve was plotted for assessing the test’s performance.

**Results:**

Mean age of the bladder cancer group was 66.41 years (standard deviation = 10.04, range = 43-82 years) and the control group was 59.67 years (standard deviation = 10.44, range = 38-77 years). All bladder cancers were of the urothelial histotype, with the following pT distribution: pTa/papillary urothelial neoplasm of low malignant potential (n = 19; 28.4%), Primary tumor (pT) in situ (n = 4; 6%), pT1 (n = 7; 10.4%), and pT≥2 (n = 7; 10.48%). Mean AHNAK2 levels were higher in bladder cancer patients 49.08 pg/mL (standard deviation = 114.91) compared to controls 5.28 pg/mL (standard deviation = 6.65), *P* < .05). Significant differences were noted between non-invasive bladder cancer (n = 23; mean = 7.14 pg/mL; standard deviation = 7.26) and invasive bladder cancer (n = 14; mean = 117.99 pg/mL; standard deviation = 168.08) and between non-muscle invasive bladder cancer (mean = 23.19 pg/mL; standard deviation = 66.93) and muscle-invasive bladder cancer (mean = 160.05 pg/mL; standard deviation = 199.65) (*P* < .001). The result of the assays was given as follows: sensitivity: 64.19%, specificity: 66.67%, positive predictive value: 22.07%, negative predictive value: 92.37%, area under curve: 0.695, and 95% CI: 0.57-0.82.

**Conclusion:**

AHNAK2 protein could be used as bladder cancer surveillance biomarker. The inclusion of AHNAK2 levels in stratification nomograms might reduce the number of unnecessary cystoscopies.

**Main Points**

- AHNAK2 urine protein levels are elevated in patients with bladder cancer.
- There is a positive correlation between AHNAK2 concentration and tumor depth.
- The incorporation of AHNAK2 urine levels into the patient’s risk-stratification nomograms might reduce the number of unnecessary cystoscopies.

## Introduction

With an estimated 550 000 new cases and 200 000 deaths, bladder cancer (BC) ranks as the 10th most common malignancy in 2020 worldwide.^1^ According to GLOBOCAN (Global Cancer Statistics), it is estimated that by 2040, the incidence and mortality of this neoplasm will double due to increased life expectancy, persistent smoking habits, and the growing adult population.^2^

Based on the depth of infiltration within the bladder wall, approximately 75% of the newly diagnosed patients fall into the non-muscle invasive bladder cancer (NMIBC) group (pTis, pTa, pT1), the remainder being classified as muscle-invasive (MIBC) group (pT≥2).^3^ While NMIBC has a favorable 5-year survival rate of 96%,^4^ its risk of progression rate could be as high as 40%.^5^ For this reason, patients with NMIBC require regular monitoring, which causes a serious economic burden and makes it an expensive malignancy to treat.^6^ On the contrary, MIBC portends a poorer prognosis, with declining 5-year survival rates of 60%, 37%, and 6% for localized, regional, and metastatic disease, respectively.^4^

Although non-invasive imaging modalities and urinary cytology (UC) are included in the BC diagnostic algorithm, cystoscopy coupled with biopsy of the suspected lesions remains the gold standard.^3^ Indeed, guidelines recommend that every hematuria patient irrespective of age needs to undergo a cystoscopy for BC detection^7^ and that cystoscopy for BC surveillance should be repeated quarterly and biannually for the first 2 years, respectively, and annually thereafter.^3^ Nonetheless, this is an invasive procedure, associated with severe discomfort and anxiety.^8^ While UC is sensitive to high-grade lesions, it has limited diagnostic accuracy in low-grade bladder tumors.^9^ Moreover, its interpretation is affected by various factors including collection procedure, low cellular yield, the presence of urinary infections, and interobserver variability.^10^

To circumvent this issue, the United States Food and Drug Administration (FDA) has approved 6 urinary tests as adjunct tools for BC diagnosis and surveillance: Polymedco’s BTA stat and BTA TRAK, Matritech’s NMP22 enzyme-linked immunosorbent assay (ELISA), Alere’s NMP22 BladderChek Test, Scimedx’s uCyt, and UroVysion (Abbott Molecular, Illinois, USA). However, due to false-positive results related to inflammatory conditions, and the technical requirements, none of these have been implemented in practice.^11^

The AHNAK2 gene located on chromosome 14q32 encodes 600-kDa protein which is an integral part of the AHNAK protein family. Apart from its physiologic role, this relatively newly discovered protein has been identified in several malignancies including uveal melanoma, renal clear-cell carcinoma, pancreatic ductal carcinoma, and papillary thyroid carcinoma.^12^

To the author’s knowledge, ELISA-based investigations for targeting AHNAK2 nucleoprotein in urine samples from BC patients have not been conducted yet.

The primary aim of this pilot study was to quantitatively measure the AHNAK2 protein in the urine of patients with BC. The secondary aim of the study was to determine the diagnostic performance of this assay and relate our findings to literature data for the conventional UC and FDA-approved biomarkers.

## Materials and Methods

### Study Population

This prospective case–control study enrolled 67 participants recruited at the University Urology Clinic “Mother Theresa,” Skopje, North Macedonia, between January and March 2019. The research was approved by the Institutional Medical Ethics Committee for Human Studies (registration number 03-5019/4) and all participants aged > 18 years signed informed written consent.

The BC group consisted of 37 patients with histologically proven BC. This group neither includes patients with upper urothelial tract malignancies, and renal tumors nor cases whose histopathological analysis was inconclusive due to tissue scantiness. Radio imaging exams in this group did not document upper urothelial or renal malignancies at the end of the study period. The microscopic interpretation was performed by 2 experienced pathologists who were blinded to the AHNAK2 urine measurements. In case of discrepancy, the final pathology diagnosis was reached by consensus. Bladder malignancies were classified and staged according to the latest World Health Organization classification system, and a 2-tiered system (low grade versus high grade) was implemented for BC grading.^13^

The control group consisted of 30 patients with histologically proven benign lesions or with no indication of BC, upper urothelial tract, or renal neoplasms, during the follow-up period, obtained from the patient medical charts. Patients with malignancies, except for basal cell cancer, were excluded from the study.

The reference standard for assessing the sensitivity (SN), specificity (SP), positive predictive value (PPV), and negative predictive value (NPV) of the test was derived from a combination of clinical, histological, and follow-up data. The investigators were blinded to each other’s results.

In February 2022, we searched the database at the Ministry of Health to evidence eventual BC malignancy within the control group.

### Urine Sample Collection

Mid-morning voided urine samples of 15 mL were collected in sterile containers from all patients before cystoscopy or surgery and centrifuged at 1000 g for 20 minutes. The resulting supernatant was aliquoted and immediately stored at −80°C until further analysis.

### Enzyme-Linked Immunosorbent Assay for the Measurement of AHNAK2 Urine Levels

Urine samples were vortexed at room temperature and centrifuged at 12000 g before use. Human AHNAK 2 quantitative sandwich ELISA kit was used (Cusabio Catalog Number. CSB-EL001480HU) to quantitatively measure AHNAK2 protein levels in all 67 voided urine specimens, according to the manufacturer’s protocol.^14^ The absorbance was measured with an ELISA plate reader, at a wavelength of 450 nm, within 5 minutes after the procedure. AHNAK2 concentration in each sample was read from the standard curve and expressed in pg/mL.

### Statistical Analysis

The gathered data were interpreted using the Statistica for Windows 8.0 software package 8.0 (StatSoft Inc. USA) and Statistical Package for the Social Sciences 25.0 software (IBM SPSS Corp.; Armonk, NY, USA). Mean and standard deviation or median and interquartile range were reported for quantitative variables, whereas frequencies and percentages were calculated for qualitative variables. The Mann–Whitney *U* test and the Student’s *t*-test were applied to assess the significance of the differences in mean AHNAK2 urine concentrations within independent groups.

The association between AHNAK2 urine levels and clinicopathological variables of the participants (gender, age, pT category, and histologic grade) was further investigated with the Spearman rank correlation test.

The receiver operating characteristic curve (ROC) was built to quantify the area under curve (AUC) in distinguishing BC patients from controls. The optimal cut-off threshold was selected at the highest point of the Youden index on the ROC curve.

The odds ratios and 95% CI were estimated using univariate and multivariate logistic regression models.

A *P* value ˂.05 was considered statistically significant.

## Results

### Study Population

The clinical and demographic characteristics of the 67 subjects are shown in Table 1. The mean age of the BC group was 66.41 years (n = 37; standard deviation (SD) = 10.04; median = 68.00; range = 43-82 years), whereas the mean age of the control group was 59.67 years (n = 30; SD = 10.44; median = 60.75; range = 38-77 years).

Histologically, all BC were of the urothelial type, with the following distribution of the pT category: 19 patients (28.4%) with pTa/papillary urothelial neoplasm of low malignant potential (PUNLMP); 4 patients (6%) with pT in situ (pTis); and 7 patients (10.4%) with each of pT1 and p≥T2, respectively. Of these, 17 cases (45.9%) were low-grade carcinomas, and 20 cases (54.1%) were classified as high-grade carcinomas.

The control group encompassed 30 cases diagnosed with benign urological diseases: urinary calculi and/or urinary tract infections (n = 13; 43.3%), benign prostate hyperplasia (n = 2; 6.67%), or non-urological conditions: inguinal hernia (n = 5; 16.67%), cholecystitis (n = 4; 13.33%), basal cell carcinoma (n = 2; 2.27%), nodular goiter (n = 2; 6.67%), and fibrocystic breast disease (n = 2; 6.67%), as evidenced by the final diagnostics.

### AHNAK2 Urine Levels in Patients with Bladder Cancer Compared to the Control Group

**Figure 1.**
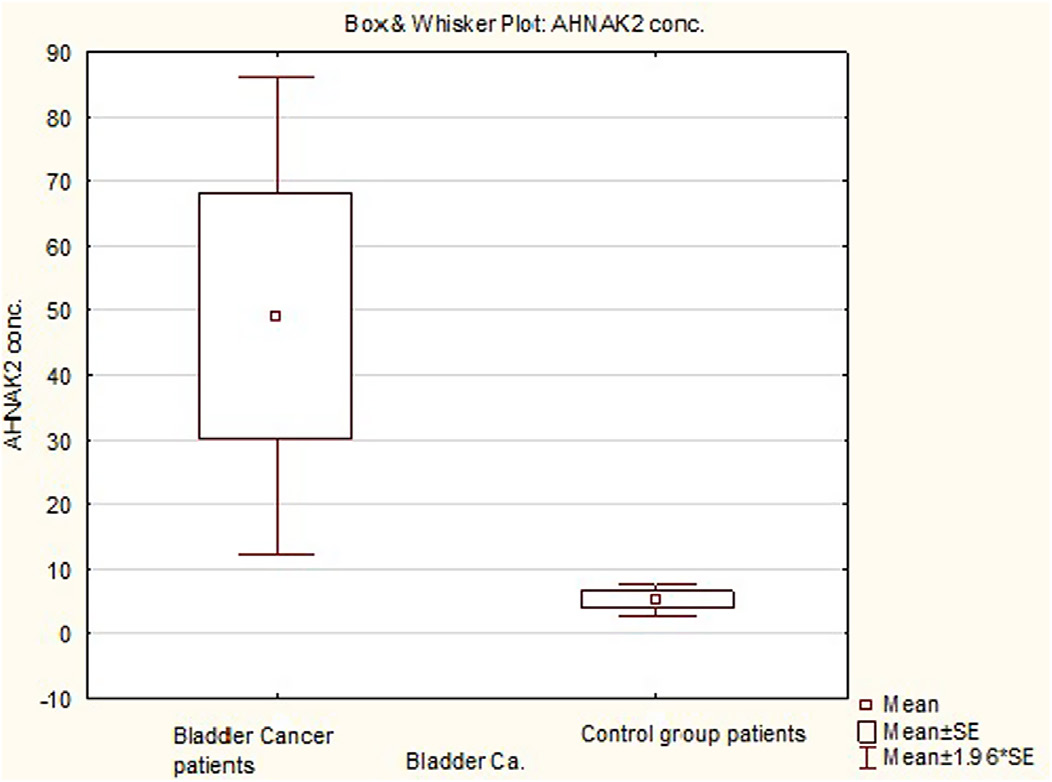
Boxplot showing AHNAK2 urinary levels in the bladder cancer group and control group.

Mean AHNAK2 urine levels were higher in BC patients 49.08 pg/mL (SD = 114.91) compared to the control group 5.28 pg/mL (SD = 6.65), (*P* < .05, Figure 1).

**Figure 2.**
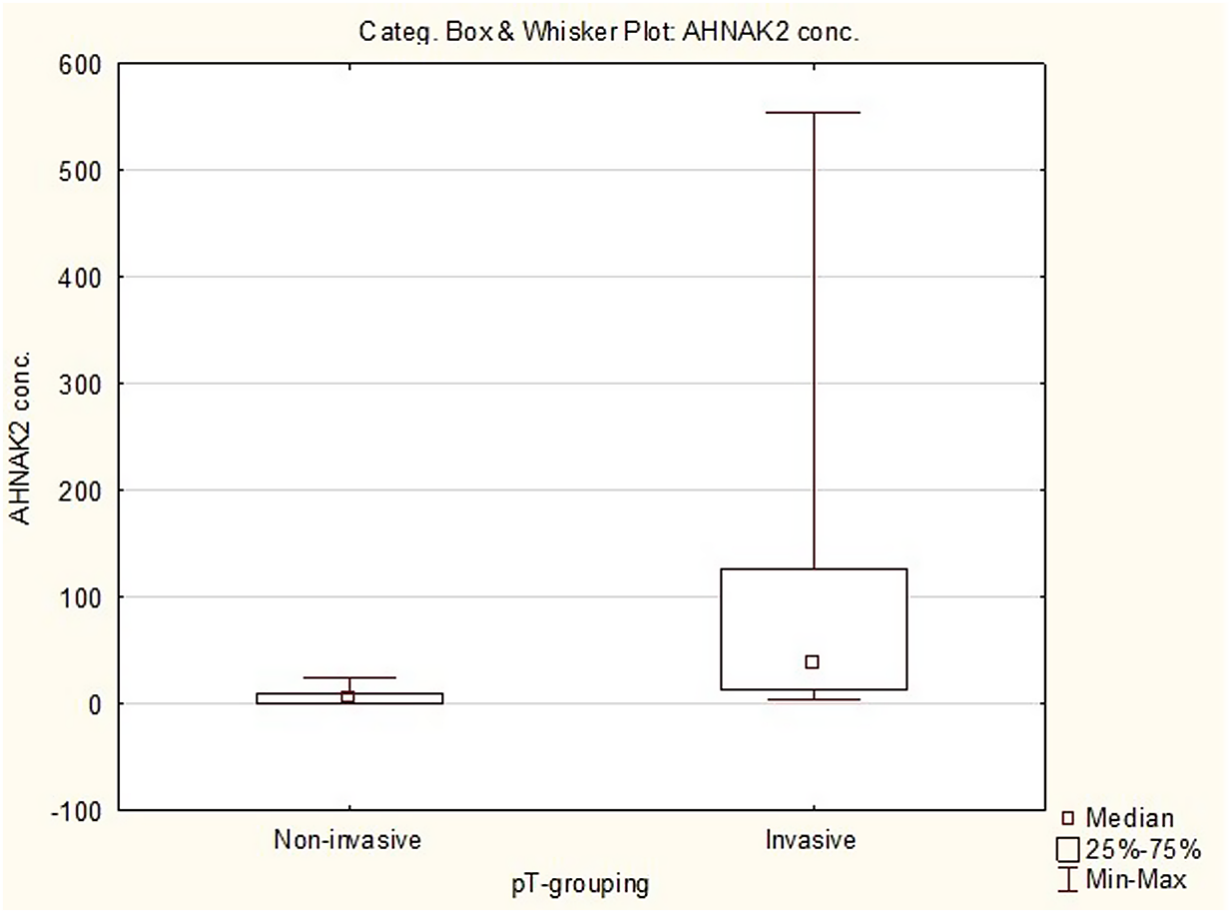
Boxplot comparing AHNAK2 urinary levels in patients with non-invasive bladder cancer (pTa/PUNLMP, pTis) and invasive (pT ≥ 1) bladder cancer.

Since the expression of AHNAK2 was increased in BC patients, we further examined these values among BC subgroups. As shown in Figure 2, we detected statistically significant differences in the mean AHNAK2 urine levels between non-invasive tumors (pTa/PUNLMP, pTis) that are confined to the basal membrane (n = 23; mean = 7.14 pg/mL; SD = 7.26) and invasive tumors (pT ≥ 1) that have infiltrated the lamina propria (n = 14; mean = 117.99 pg/mL; SD = 168.08) (*P* < .01).

**Figure 3.**
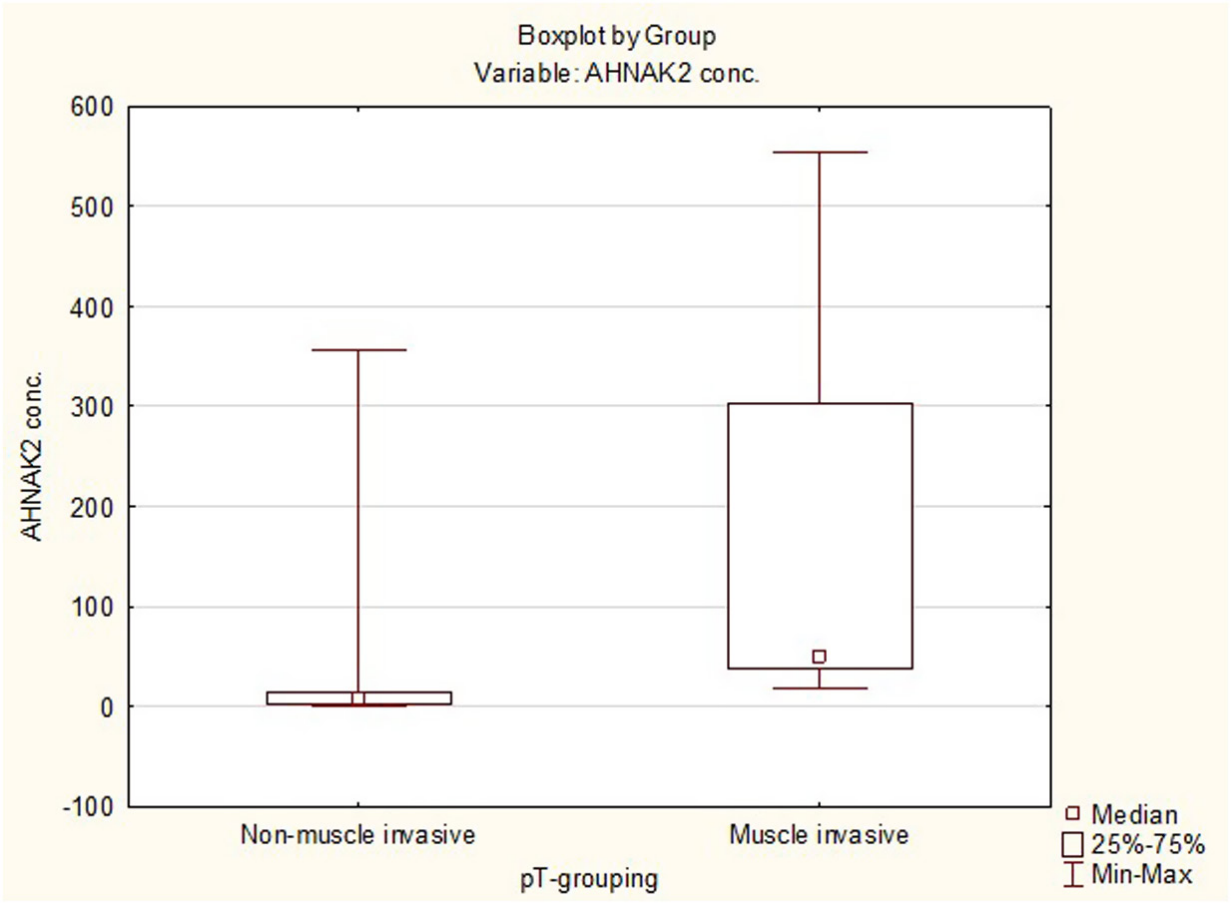
Boxplot comparing AHNAK2 urinary levels in patients with non-muscle invasive bladder cancer (pTa, pTis, pT1) and muscle-invasive bladder cancer (pT ≥ 2).

We also looked if we could discriminate against NMIBC from MIBC. Data showed significant differences in AHNAK2 concentrations between NMIBC (mean = 23.19 pg/mL; SD = 66.93) and MIBC patients (mean = 160.05 pg/mL; SD = 199.65) (*P* < .001), (Figure 3).

Meanwhile, AHNAK2 concentrations were not correlated with urinary tract infection/calculosis smoking status, history of BC, or age, (all *P* > .05) and expressed a weak correlation with macrohematuria (*P* < .05)

### Performance Characteristics

**Figure 4.**
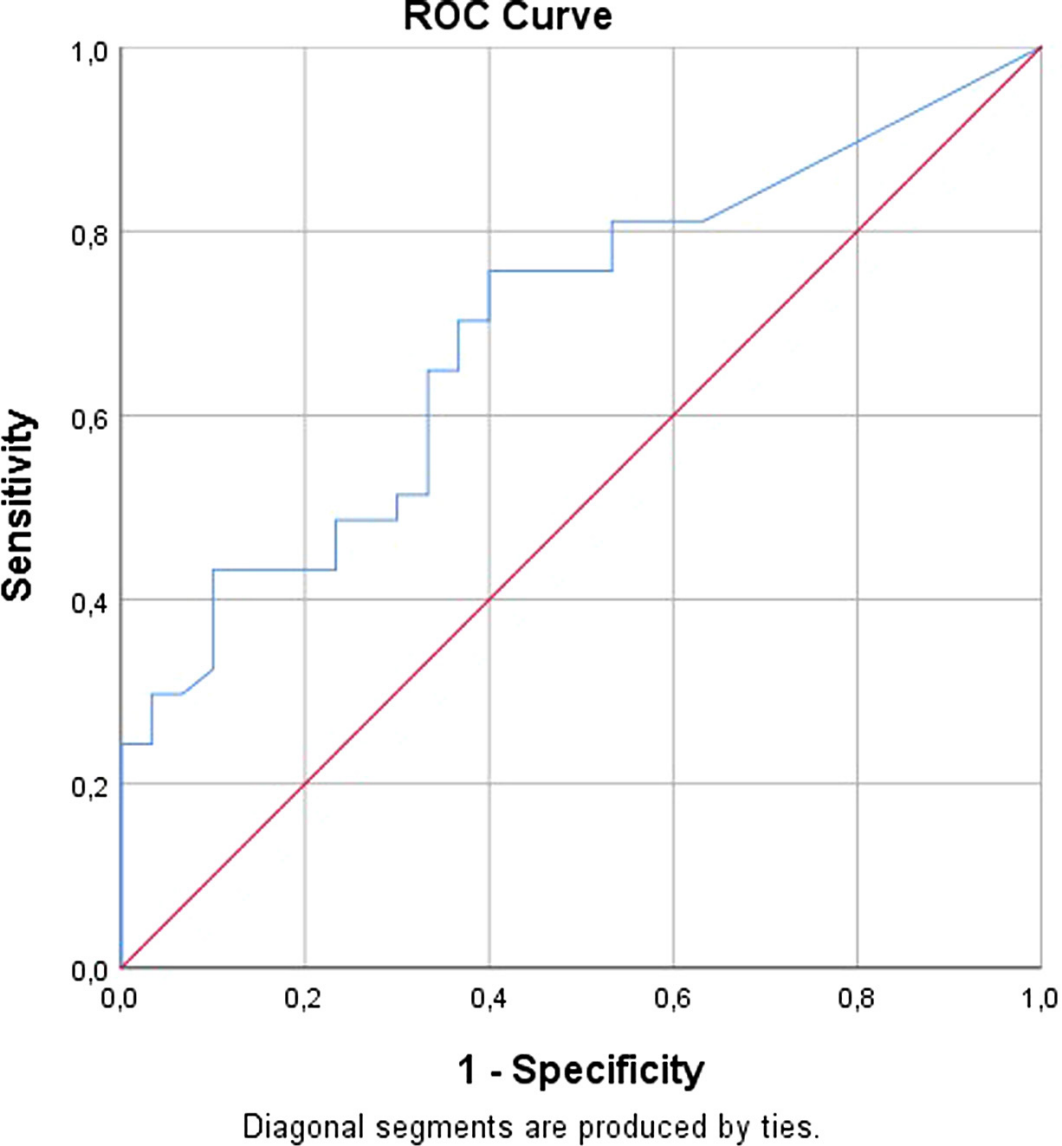
ROC curve based upon AHNAK2 urinary concentrations to determine the diagnostic characteristics. AHNAK2 urinary levels generated an AUC value of 0.695 with 64.9% sensitivity and 66.67% specificity in distinguishing bladder cancer patients (n = 37) from controls (n = 30).

We used a ROC analysis to look at the diagnostic accuracy of urine AHNAK2 concentrations for BC diagnosis. Using this method, we acquired an AUC of 0.695 for AHNAK2 (95% CI: 0.57-0.82) (Figure 4).

The cut-off value of 5.48, displayed SN of 64.9% (95% CI: 45.99-78.19), SP of 66.67% (95% CI: 45.67-82.06), with PPV of 22.07% and NPV of 92.37% (Table 2).

We further stratified BC patients according to the histologic grade. The diagnostic accuracy for low-grade tumors was: SN: 64.71%, SP: 66.67%, PPV: 22.64% and NPV: 92.61%. For high-grade tumors, the SN, SP, PPV, and NPV were 65.00%, 66.67%, 22.72%, and 92.67%, respectively (Table 3).

## Discussion

In this study, we performed ELISA analysis aiming to investigate the relationship between AHNAK2 urine concentration and BC occurrence. In this regard, we noted several significant observations. First, mean AHNAK2 concentrations in BC patients were 10-fold higher compared to the mean values of the control group. Second, these concentrations were 16.5 times greater in invasive BC as opposed to non-invasive BC. Third, MIBC patients had on average 7 times higher AHNAK2 levels in comparison to NMBIC patients. Fourth, contrary to FDA-approved ELISA protein-based tests, inflammatory conditions did not affect the assay’s performance.

Subsequently, we evaluated our findings against data from studies scrutinizing the diagnostic performance of UC and the FDA-approved biomarkers for BC diagnosis and noted mixed results.

With respect to sensitivity, we found superior results compared to UC. According to a study that combined data from 25 meta-analyses, mean sensitivity of the UC was 45.5% (SD = 23.1).^15^ Similar values of 37% (95% CI: 35%-39%) were presented in another recent review.^16^ Furthermore, our sensitivity results were comparable to those observed in the protein-based FDA biomarkers. For example, a systematic review by Chou et al^17^ demonstrated a sensitivity of 58% and 69% for the Qualitative NMP22 and Quantitative NMP22 protein tests, respectively. Wang et al^18^ explored the diagnostic performance of NMP22 Bladder Check, in 23 systematic studies and 19 studies in the quantitative meta-analysis, and noted a sensitivity of 56%. Similarly, sensitivity for both BTA stat and BTA track assays varied from 64 to 67%.^17,19^ Nevertheless, we report lower sensitivity compared to fluorescence-based UroVysion and Immunocyte probes. For instance, UroVysion test’s pooled sensitivity varied between 55% and 81%.^20^ Likewise, higher sensitivity that ranged between 50% and 85% was observed for the immunocyte test.^21^

With respect to specificity, our data did not reach the values from the above-mentioned tests. For illustration, previous research has shown that the pooled specificity for the quantitative NMP22 test was 0.77 (95% CI: 0.70-0.83),^17^ while the specificity for the qualitative NMP22 test reached 88%.^17,18^ Correspondingly, the specificity of the BTA-Stat quantitative test and BTA-Trak qualitative test ranged 75%-77% and 68%-87%, respectively.^17,18^ Based on a systematic review and meta-analysis, UroVysion test’s specificity was found to be 0.85 (95% CI: 0.76-0.91).^22^ These findings were concordant with another recent analysis, which presented specificity from 66% to 96%.^20^ Higher specificity varying from 62.8% to 78% was also detected for immunocyte test.^17,21,23^ Of note, none of these kits have surpassed UC, which has a median specificity of 94.9%.^15^

We discovered interesting findings regarding predictive values. Even though our PPV was lower, the NPV of this test was superior as opposed to the mean NPV of all other tests: BTA Stat (67.9%; SD = 17.1), UroVysion (72.4%; SD = 24.6), UC (82.6%; SD = 16.1), NMP22 (82%; SD = 16.6), and immunocyte (83.4%; SD = 21.4).^15^

Another surprising finding was observed regarding the tumor grade. We detected higher sensitivity for low-grade tumors (G1) compared to UC (10%-43%),^9^ quantitative and qualitative NMP22 test (48%; SD = 25.7), and BTA test (42.9%; SD = 5.7).^15^ The sensitivity for low-grade tumors was analogous to that of the fluorescent-based probes. In particular, the reported sensitivity of the UroVysion test varies between 40.8%^24^ and 66.5%,^15^ whereas immunocyte’s sensitivity is 67.3%.^15^ However, these tests outperformed our sensitivity for high-grade (G2/G3) lesions.^15^

As evident from the preceding paragraphs, estimates of the accuracy characteristics differ across studies. Plausible theories for these discrepancies are various research designs, non-matched controls, sample size variations, retrospective nature of the studies, and non-consecutive sample recruitment.

Of course, advanced molecular techniques and the new omics approaches have resulted in a plethora of novel biomarkers, reporting better performance characteristics for early diagnosis and surveillance of BC.^11^ However, these analyses are either costly, labor-intensive, or require diligent validation.^11^

To the best of our knowledge, few reports have investigated the diagnostic potential of the AHNAK protein family in BC. Previous investigations have observed AHNAK cytoplasmic immunohistochemistry expression in BC tissues.^25^ Additionally, Lee et al^26^ indicated that AHNAK nuclear positivity in BC cells in liquid-based cytology could reliably discriminate against them from benign urothelial lesions. Likewise, AHNAK2 protein expression was found in pT2/T3 tumors of optimal cutting temperature compound and subsequently frozen samples.^27^ Another study using the Fourier transform infrared imaging had proven that AHNAK2 immunohistochemical expression could distinguish reactive urothelial atypia from carcinoma in situ, with a sensitivity of 97% and a specificity of 69%. In the same study, the calculated sensitivity and specificity between low- and high-grade tumors were 80% and 86%, respectively.^28^ However, major drawbacks of the above-mentioned research were that these proteomic studies validated AHNAK2 protein in a tissue biopsy, while immunocytochemical studies on liquid-based cytology warrant larger cohorts.

Bladder cancer remains a major health problem. In comparison to other genitourinary malignancies, the 5-year survival rate has not improved in the last 3 decades.^29^ Even more, the COVID-19 pandemic has led to dramatic diagnostic and treatment delays in BC patients.^11^ On the other hand, due to significant differences in treatment approaches and survival rates between NMIBC and MIBC patients, early detection and close monitoring of patients with already diagnosed BC are critical.^3^ One possible solution toward improving BC survival outcomes is the application of a fast, accurate, and inexpensive non-invasive biomarker.

Some advantages are worth mentioning in this report. First, increased AHNAK2 protein urine levels could alert both the urologist and the pathologist to the possibility of BC. Unlike dichotomized tests, the quantitative character of this assay might prove useful in better triaging individuals who require additional cystoscopy and in the prediction of high-risk tumors. This could also be helpful in circumstances where histopathological interpretation is hampered by the tissue’s scantiness or cauterization artifacts. Second, this is an easy-to-perform ELISA assay that can be run in most hospital laboratories and can deliver results within 3 hours. Third, in contrast to other molecular techniques, this assay is affordable, without the need for specialized technical expertise and maintenance. Fourth, we collected fresh mid-morning urine to prevent significant proteolysis and urine contamination, thus reducing the pre-analytical bias. Fifth, since a high NPV biomarker is required to avoid cystoscopies,^21^ the strong NPV of this test, for both low-grade and high-grade tumors, makes it convenient in a BC surveillance setting. This would be beneficial for patients and the healthcare system. Lastly, our report adds to the existing link between AHNAK2 and BC, and importantly, it contributes to its potential applicability as non-invasive biomarker.

The current study has the following limitations. Foremost, it delivers data from a single-institution center and the study power is low due to the small sample size. Another limitation of the study is the omission of individuals with renal and upper urinary tract malignancies, which could have affected the results. Consequently, these results should be explored with great caution. Nevertheless, it could initiate more extensive, multi-institutional research, encompassing a larger group of patients. Additionally, we acknowledge the selection bias, since the control group included patients treated for both urological and non-urological diseases. This implies that the selected participants may not have accurately represented the source population, and special consideration should be taken when interpreting the findings. Still, ultrasound examinations during control subjects’ recruitment did not reveal urinary bladder, upper urinary tract, or renal neoplasms, and their health status based on the Ministry of Health software’s database was reaffirmed at the end of the follow-up period. Finally, albeit we achieved high NPV and improved sensitivity for low-grade tumors, the overall sensitivity and specificity were fair. The likely explanation is that we did not take into account the urinary creatinine volume, urine specific gravity, and osmolality, which serve as important normalization components for the effect of urine hydration on AHNAK2 concentrations.^30^ However, we anticipate that the incorporation of these analyses will improve the test performance.

Although ELISA-based AHNAK2 urine analysis cannot completely replace cystoscopy, our findings suggest that this non-invasive method could represent a promising adjunct tool for BC detection. We believe that the inclusion of these data into the risk-stratification nomograms, combined with UC, might correctly identify patients at-risk, who require further investigations while minimizing invasive procedures in low-risk individuals. This approach might eventually contribute to the optimization of health care resources. Larger, prospective trials in real-life clinical scenarios with a consecutive sampling design and matched controls are needed to further investigate these findings.

## Figures and Tables

**Table 1.** Clinicopathologic Characteristics of the Study Population

	Bladder Cancer Group, N = 37 (%)	Control Group, N = 30 (%)
Median age (range, years)	68 (43-82)	60.5 (38-77)
Male-to-female ratio	5.17	1
Smoker/former smoker		
Yes	34	19
No	3	11
Gross hematuria		
Yes	18	5
No	19	25
Pathologic T category		n/a
pTa/PUNLMP	19 (28.4)	
pTis	4 (6)	
pT1	7 (10.4)	
pT ≥ 2	7(10.4)	
Histologic grade		n/a
Low	17 (45.9)	
High	20 (54.1)	
History of bladder cancer		n/a
Yes	18	
No	19	
Benign urological diseases	n/a	
UTI/calculosis		13 (43.3)
BPH		2 (6.67)
Benign non-urological diseases	n/a	
Inguinal hernia		5 (16.67)
Cholecystitis		4 (13.33)
Basal cell carcinoma		2 (6.67)
Nodular goiter		2 (6.67)
Fibrocystic breast disease		2 (6.67)

PUNLMP, papillary urothelial neoplasm of low malignant potential; pTis, carcinoma in situ; n/a, not applicable; UTI, urinary tract infections; BPH, benign prostatic hyperplasia.

**Table 2.** Performance of AHNAK2 Enzyme-Linked Immunosorbent Assay Urine Test at Cut-Off Value of 5.48 pg/mL for the Detection of Bladder Cancer

	Cut-Off Value	Diagnostic Characteristics % (95% CI)	
Bladder cancer group versus control group	5.48	True positive, n =24	SN 64.9% (95% CI: 45.99-78.19)
		True negative, n =20	SP 66.67% (95% CI: 45.67-82.06)
		False positive, n =10	PPV 22.07% (95% CI: 3.14-9.00)
		False negative, n = 14	NPV 92.37% (95% CI: 97.23-98.95)

SN, sensitivity; SP, specificity; PPV, positive predictive value; NPV, negative predictive value.

**Table 3.** Overall Sensitivity, Specificity, Positive Predictive Value, and Negative Predictive Value of AHNAK2 according to tumor grade, with a cut-off value of 5.48

Tumor Grade	Cut-Off Value	Diagnostic Characteristics % (95% CI)
Low-grade	5.48	SN 64.71 (38.33-85.79)
		SP 66.67 (47.19-82.71)
		PPV 22.64 (13.65-35.14)
		NPV 92.61 (86.25-96.16)
High-grade		SN 65.00 (40.78-84.61)
		SP 66.77 (47.19-82.71)
		PPV 22.72 (13.90-34.87)
		NPV 92.67 (86.85-96.03)

SN, senstitivity; SP, specificity; PPV, positive predictive value; NPV, negative predictive value.
